# Tweaking Polybia-MP1: How a Lysine-Histidine Swap Redefines Its Surface Properties

**DOI:** 10.3390/pharmaceutics17101287

**Published:** 2025-10-02

**Authors:** Kenneth M. F. Miasaki, Bibiana M. Souza, Mario S. Palma, Natalia Wilke, João Ruggiero Neto, Dayane S. Alvares

**Affiliations:** 1 Department of Physics, IBILCE, UNESP—São Paulo State University, São José do Rio Preto 15054-000, SP, Brazil; kenneth.miasaki@unesp.br; 2 Department of Basic and Applied Biology, Institute of Biosciences, UNESP—São Paulo State University, Rio Claro 13506-900, SP, Brazil; bibiana.souza@unesp.br (B.M.S.); mario.palma@unesp.br (M.S.P.); 3 Centro de Investigaciones en Química Biológica de Córdoba (CIQUIBIC), CONICET, Haya de la Torre y Medina Allende, Ciudad Universitaria, Córdoba X5000HUA, Argentina; natalia.wilke@unc.edu.ar; 4 Departamento de Química Biológica Ranwel Caputto, Facultad de Ciencias Químicas, Universidad Nacional de Córdoba, Córdoba X5000HUA, Argentina

**Keywords:** membrane organization, pH-responsive peptide, lipid monolayer

## Abstract

**Background/Objectives:** Polybia-MP1 (MP1) exhibits antimicrobial and anticancer properties. To improve selectivity toward acidic tumor microenvironments, we designed HMP1, a histidine-substituted analog of MP1, aiming to introduce pH-responsive behavior within physiological and pathological pH ranges. **Methods:** HMP1 was synthesized by replacing all lysine residues in MP1 with histidines. We characterized its surfactant properties and interactions with lipid monolayers composed of DPPC under varying pH and ionic strength conditions. Langmuir monolayer experiments were used to evaluate peptide-induced morphological changes and lipid packing effects at physiologically relevant lateral pressures. **Results:** HMP1 displayed pH-dependent activity between pH 5.5 and 7.5, inducing significant morphological reorganization of lipid domains without reducing the condensed phase area. Ionic strength modulated these effects, with distinct behaviors observed at low and physiological saline conditions. HMP1 preferentially interacted with cholesterol-enriched membranes, while MP1 did not induce comparable effects under the same conditions, as previously reported, at physiological lateral pressures. HMP1 also exhibited non-hemolytic properties and lower cytotoxicity compared to MP1. **Conclusions:** The lysine-to-histidine substitution conferred pH sensitivity to HMP1, enabling selective modulation of membrane organization based on lipid composition, packing, pH, and ionic environment. These findings highlight HMP1’s potential in targeted therapeutics and pH-responsive drug delivery systems.

## 1. Introduction

Antimicrobial peptides (AMPs) constitute a diverse class of biologically active molecules that play essential roles in innate immunity across multiple species [1,2,3]. Beyond their natural defense functions, AMPs have attracted considerable interest as potential therapeutic agents due to their broad-spectrum antimicrobial activity and, in some cases, selective cytotoxicity against cancer cells [3,4,5].

Among these, Polybia-MP1 (MP1), a cationic peptide derived from the venom of the wasp *Polybia paulista*, has been extensively studied for its potent bactericidal effects against Gram-positive and Gram-negative bacteria, as well as its ability to inhibit the proliferation of various cancer cell lines [5,6,7,8,9,10]. Its relatively small size, amphipathic helical structure, and lysine-rich composition make MP1 an attractive scaffold for rational design, as subtle modifications can result in measurable changes in selectivity and activity. In addition, our group has extensively characterized MP1’s structure, functional properties, and membrane interactions over the past decade, thereby providing a strong framework for implementing targeted structural modifications aimed at optimizing its therapeutic potential [11,12,13,14,15,16,17,18,19,20].

Studies have revealed that in some cancer tissues, the extracellular milieu is one or two pH units below the physiological healthy counterpart [21,22,23]; consequently, the tumor microenvironment has recently attracted attention. Findings in the literature have shown that cell-dependent pH changes can be tuned between intracellular and extracellular and can be crucially associated with tumor growth, proliferation metastasis, and resistance to treatment [24,25,26]. Recognizing the significance of these pH gradients and seeking to develop a peptide responsive to such subtle variations, we designed a modified version of MP1, termed HMP1, by substituting its three lysine residues with histidines. This Lys→His substitution introduces pH sensitivity due to the histidine side chain’s pKa near physiological pH, allowing us to probe how protonation dynamics influence peptide–membrane interactions in acidic tumor-like environments. Importantly, this engineered peptide is both non-hemolytic and non-cytotoxic, supporting its potential for biomedical applications [27].

Understanding how such peptides behave at interfaces is crucial not only to elucidate their mechanisms of action but also for advancing the rational design of membrane-active peptides with optimized properties for membrane biophysics, drug delivery, biomaterials engineering, and therapeutic applications. The air–water interface, in particular, offers a simplified yet informative model for investigating peptide-surface interactions and provides valuable insights for the development of peptides with optimized interfacial and membrane activity [28,29].

Lipid monolayers at this interface have proven to be valuable systems for gaining molecular-level insights into peptide-lipid interactions. In previous work, we explored how MP1 influences the phase behavior of dipalmitoylphosphatidylcholine (DPPC) monolayers under different pH and ionic strength conditions, employing Brewster angle microscopy (BAM) to analyze domain morphology [14]. Our findings indicated that MP1 co-crystallized with DPPC into branched domains exclusively in pure water subphases. In contrast, at high salt concentrations or extreme pH values, MP1 formed less compact films and was excluded from lipid domains, suggesting that electrostatic interactions, likely mediated by salt bridges between anionic aspartic acids and cationic lysines, promote peptide aggregation and co-crystallization.

Given this evidence, the present study investigates how pH and ionic strength modulate the interfacial properties of the histidine-substituted peptide HMP1 and its interaction with DPPC using Langmuir monolayers as model membranes. DPPC is a well-characterized saturated phospholipid with liquid-expanded (LE) and liquid-condensed (LC) phases and a high transition temperature (41.5 °C) [30]. The choice of DPPC as the model lipid in this study provides a well-defined platform for the visual and quantitative assessment of peptide-induced effects in ordered lipid environments. In this study, no new experiments were performed with MP1; the results obtained for HMP1 were compared with previously published data for MP1 from Alvares et al. [14]. Here, we investigate how a histidine-rich peptide modulates the organization, morphology, and stability of DPPC monolayers under varying conditions of peptide concentration, pH, and ionic strength. This approach enables a comprehensive evaluation of the peptide’s impact on the structural properties of model membranes.

## 2. Materials and Methods

### 2.1. Chemicals and Reagents

1,2-Dipalmitoyl-sn-3-glycerophosphocoline (DPPC), 1-palmitoyl-2-oleoyl-glycero-3-phosphocholine (POPC), cholesterol (Chol), and 1-palmitoyl-2-6-[(7-nitro-2-1,3-benzoxadiazol-4-yl)amino]hexanoyl-sn-glycero-3-phosphocholine (NBD-PC) were purchased from Avanti Polar Lipids (Alabaster, AL, USA). Sodium citrate trisodium salt, sodium borate, monobasic sodium phosphate, and sodium chloride (NaCl) were obtained from Sigma-Aldrich. Sodium hydroxide (NaOH), hydrochloric acid (HCl), chloroform, and methanol were obtained from Merck (Darmstadt, Germany). All reagents were of analytical grade. Ultrapure water (Millipore, Burlington, MA, USA; 18 MΩ·cm) was used for the preparation of the subphases. The buffer (CBP) contained 1 mM sodium citrate, sodium borate, and sodium phosphate, plus 150 mM NaCl, at pH 5.5, 6.5, or 7.5.

### 2.2. Peptide Synthesis

The peptide HMP1 (${\mathrm{IDWHHLLDAAHQIL-NH}}_{2}$) was synthesized by stepwise manual solid-phase peptide synthesis [31,32,33], using N-9-fluorophenylme-thoxy-carbonyl (Fmoc) chemistry with Novasyn TGS resin from Novabiochem (Merck KGaA, Darmstadt, Germany) with a loading capacity of 0.26 nmol/g, as specified by the manufacturer. The removal of the peptide-resin complexes was performed with a mixture of trifluoroacetic acid/1,2 ethanedithiol/anisole/phenol/water (82.5:2.5:5:5:5 by volume), using 10 mL/g of complex at room temperature for 2 h. The resin was then filtered off, and the filtrate was treated with cold anhydrous diethyl ether (acquired from Sigma; St. Louis, MO, USA) at 4 °C to precipitate the crude peptide. The resulting pellet was collected by centrifugation at 1000× *g* for 15 min at room temperature, resuspended in water, and purified by reverse-phase high-performance liquid chromatography (RP-HPLC) using a semi-preparative C18 column (250 × 10 mm, 5 µm; Shiseido Co., Ltd., Tokyo, Japan) at a flow rate of 2 mL/min under isocratic conditions (46% *v*/*v* acetonitrile in water containing 0.1% *v*/*v* trifluoroacetic acid). Peptide purity was determined by analytical RP-HPLC at 220 nm, with the reported 96% purity corresponding to the area under the main peptide peak relative to all detectable peaks in the chromatogram. The molecular weight and identity of the purified peptide were confirmed by electrospray ionization mass spectrometry (ESI-MS).

### 2.3. Adsorption onto Clear Interface

The adsorption of the peptide to the air–liquid interface was assessed in experiments at constant area using a magnetically stirred circular Teflon well (Kibron Inc., Helsinki, Finland) DeltaPi tensiometer (Kibron, Helsinki, Finland) as detailed in [29]. The change in surface pressure (Δπ) was recorded using a Wilhelmy wire connected to a microbalance (DeltaPi, Kibron Inc.). Surface activity curves (Δπ as a function of peptide concentration ($C_{p}$) in the subphase at different pH) were fitted using a non-linear least-squares regression, as described by Alvares et al. [16]. From these fits, the surface excess concentration of the peptides (Γ) wa s calculated using the Gibbs adsorption equation:

(1) $$\Gamma =\frac{\Delta \pi }{RT\;\Delta ln(C_{p})}$$

where R is the gas constant and T the temperature. Using the peptide’s maximum surface excess concentration ($\Gamma_{max}$), the molecular area ($A_{ads}$) can be calculated as follows:

(2) $$A_{ads}=\frac{1}{N\;\Gamma_{max}}$$

where N is Avogadro’s constant. All experiments were conducted at 20 °C in water and CBP buffer at the desired pH (5.5, 6.5, or 7.5). Standard deviations represent the results of at least three independent experiments. Data analysis was performed using OriginPro, Version 2024b (OriginLab Corporation, Northampton, MA, USA).

### 2.4. Insertion into Lipid Monolayer

The insertion of HMP1 into POPC, 3POPC:1Chol, and DPPC lipid monolayers was evaluated using constant-area experiments with a DeltaPi instrument (Kibron Inc., Helsinki, Finland). Monolayers were prepared by spreading a lipid solution (chloroform:methanol, 2:1) onto the surface of a buffer solution until reaching a surface pressure of 30 mN/m. Then, the peptide was injected into the subphase under continuous stirring to a final concentration of 1.25 µm. Peptide insertion was monitored by the change in surface pressure over time. All experiments were conducted at 20 °C, and standard deviations were calculated from at least three independent measurements.

### 2.5. Compression Isotherms

Surface pressure (π) of peptides, lipids, or lipid/peptide mixtures was measured using a platinum Wilhelmy plate. Compression isotherms of monolayers [29] were continuously recorded using a KSV Minitrough (243 cm^2^) apparatus (KSV, Helsinki, Finland), filled with either ultrapure water or CBP buffer at the desired pH.

#### 2.5.1. Preparation of Pure Peptide Monolayer

Pure peptide monolayers were prepared by spreading a peptide solution in methanol onto the surface of water or CBP buffer subphase using a Hamilton microsyringe (Reno, NV, USA). Compression isotherms were obtained at a compression rate of 0.2–$0.5\;{Å}^{2}{\mathrm{molecule}}^{-1}s^{-1}$ (equivalent to 7–20 mm/min).

#### 2.5.2. Lipids and Lipid-Peptide Premixed

Small drops of lipid or lipid-peptide premixed at the desired molar ratio and dissolved in chloroform/methanol (2:1 (*v*:*v*)), were spread directly onto the air-liquid interface. After solvent evaporation (≈10 min), the monolayers were compressed at a rate of 7 mm/min at 20 °C. Monolayers doped with 0.5 mol% of NBD-PC were additionally monitored by fluorescent microscopy using a trough with a glass window mounted on the stage of an Olympus IX71 inverted microscope (Olympus, Tokyo, Japan) equipped with a CCD camera. Standard deviations of each isotherm were below 3%, based on at least three independent experiments. Fluorescence micrographs were analyzed using the NIH open-source software FIJI/ImageJ (National Institutes of Health, Bethesda, MD, USA). The mixing behavior of lipids and peptides can be assessed by determining the excess area ($A_{ex}$), which compares the experimental mean molecular area of the mixture ($A_{12}$) with that of an ideal mixture, calculated as,

(3) $$A_{ex}=A_{12}-\left(A_{1}X_{1}+A_{2}X_{2}\right)$$

where $X_{1}$ and $X_{2}$ represent the molar fractions of component 1 (lipid) and component 2 (peptide), respectively, and $A_{1}$ and $A_{2}$ denote their corresponding mean molecular areas at a specified surface pressure [34]. The value of $A_{12}$ was recalculated taking into account the peptide amount.

The elastic behavior of the film was also investigated through the compressibility modulus, $C_{s}^{-1}$ calculated as,

(4) $$C_{s}^{-1}=-A{\left(\frac{\partial \pi }{\partial A}\right)}_{\;T}$$

where *A* represents the area per molecule, and the derivative corresponds to the slope of the isotherm at a given surface pressure π [35]. The compressibility modulus, which is the inverse of compressibility, $C_{s}$, is calculated from surface pressure–area (π – *A*) isotherms. This parameter can be used to identify phase transition regions and distinct phases within the isotherms. Data analysis was performed using OriginPro, Version 2024b (OriginLab Corporation, Northampton, MA, USA).

## 3. Results and Discussion

### 3.1. Interfacial Adsorption and Peptide Packing Properties

#### 3.1.1. Influence of Subphase pH on Monolayer Collapse Pressure and Packing

**Figure 1:**
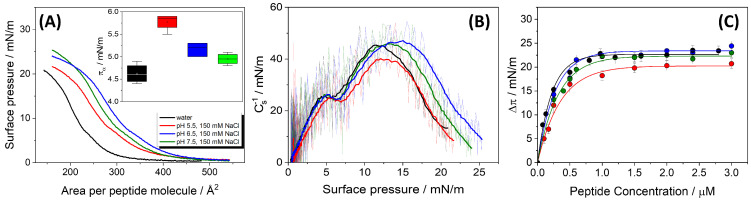
(**A**) Surface pressure–mean molecular area (π–*A*) compression isotherms of pure HMP1 films spread on water (black) or CBP buffer containing 150 mM NaCl at pH 5.5 (red), 6.5 (blue), or 7.5 (green). Inset: Surface pressure at the first inflection point ($\pi_{\alpha }$) in the π–*A* isotherm. The value of $\pi_{\alpha }$ obtained for water was significantly different from those at pH 5.5 and 6.5 (*p* < 0.05), as determined by Tukey’s test following ANOVA. (**B**) Compressibility modulus, surface pressure curves ($C_{s}^{-1}$-π) for HMP1 films, derived from the isotherms shown in panel (A). (**C**) The maximum change of surface pressure (Δπ) as a function of peptide concentration ($C_{p}$) injected on pure water subphase (black) or CBP buffer at pH 5.5 (red), 6.5 (blue), or 7.5 (green). The data represent the average ± standard error of at least three independent measurements. The continuous lines represent the non-linear least-squares regression analysis. All experiments were registered at T = 20 °C.

Surface pressure–area (π–*A*), and compression modulus isotherms of HMP1 Langmuir monolayers are presented in Figure 1A,B. The π–*A* isotherms of the pure peptide demonstrate that HMP1 is capable of forming stable monolayers, two distinct transitions are observed in the isotherms (Figure 1A). The first transition ($\pi_{\alpha }$) probably corresponds to a conformational rearrangement of the peptide at the air–water interface. This rearrangement is evident as a minimum in the compressibility modulus plot (Figure 1B) and is absent in the monolayers of MP1 [14], suggesting that the histidine residue (His) is involved in the process. This bulky residue probably adopts a different orientation upon compression. Reorientation occurs at a surface pressure that depends on pH and salt presence (inset in Figure 1A), suggesting that both histidine and anionic residues are involved in modulating the supramolecular structure of the monolayer. Notably, this rearrangement is not detectable by fluorescence microscopy with the NBD-PC probe, indicating that the reorganized peptide interface does not display altered affinity for the fluorescent label.

The second transition was attributed to monolayer collapse ($\pi_{c}$). These transitions exhibited notable sensitivity to the subphase pH and ionic strength (Figure 1A and Table 1). Although 150 mM NaCl reflects physiological ionic strength, experiments were also performed on pure water subphases to isolate the intrinsic interfacial behavior of the peptide in the absence of salt. This comparison allows the identification of specific effects induced by ionic strength, particularly regarding peptide conformation, packing density, and monolayer stability, providing a more comprehensive understanding of the role of electrostatic interactions at the interface.

The ionization state of HMP1 under these pH conditions was estimated using the pKa values of its ionizable groups: 4.0 (Asp), 6.5 (His), and 8.0 (N-terminal amine), assuming negligible intramolecular electrostatic coupling [36]. At pH 5.5, approximately 91% of histidine residues are protonated, while aspartic acid residues remain deprotonated, and the N-terminus is fully protonated, resulting in a calculated net positive charge of approximately +1.79. At pH 7.5, acid residues remain deprotonated and histidine is largely neutral, generating a net charge of approximately –0.97. At pH 6.5, the net charge is close to neutrality, with a value of approximately +0.47. Estimated net charges and the contribution of each residue are shown in Table 2.

Interestingly, the collapse pressure ($\pi_{c}$) of the HMP1 monolayers exhibits a greater sensitivity to variations in the pH of the subphase than to changes in the mean molecular area ($A_{p}$). As pH increases from 5.5 to 7.5, $\pi_{c}$ increases slightly from 18.5 ± 0.2 to 21.7 ± 0.2 mN/m. These results suggest that $\pi_{c}$ is mainly determined by the bulk pH rather than ionic strength, as similar collapse pressures are observed in both pure water and NaCl at pH 5.5. In particular, $\pi_{c}$ increases as the pH increases and the histidine residues become neutral, suggesting that the peptide’s ability to stabilize the interface is enhanced when these residues are not charged.

In contrast to $\pi_{c}$, the mean molecular area at collapse ($A_{p}$) exhibits a non-linear dependence on pH. At pH 5.5, $A_{p}$ is 351 ± $8\;{Å}^{2}$, which then increases to a maximum of 370 ± $9\;{Å}^{2}$ at pH 6.5, before decreasing back to 351 ± $1\;{Å}^{2}$ at pH 7.5. This non-monotonic behavior indicates that the packing density at the point of collapse is not directly proportional to pH or stability.

It is important to note that, in the absence of salt, the positive charge of the monolayer is not screened by salt ions, but rather by hydroxide ions present in the subphase. As a result, these anions accumulate at the interface, leading to a concomitant depletion of protons and an increase in the local interfacial pH. Although a similar effect occurs at high ionic strength, the impact is markedly attenuated due to the efficient screening provided by the salt ions in solution. To estimate the local pH under the experimental conditions in pure water, we employed the Gouy–Chapman model, widely applied in the context of peptide/lipid membrane interaction [17,18,37,38]. According to this model, the surface pH ($pH_{\mathrm{sf}}$) differs from the bulk pH ($pH_{\mathrm{sb}}$) as follows:

(5) $$pH_{\mathrm{sf}}=pH_{\mathrm{sb}}+\frac{F\psi_{0}}{2.3RT}$$

where *F* is the Faraday constant, $\psi_{0}$ is the double layer potential at the interface, *R* is the gas constant, and *T* is the absolute temperature [39,40]. In this context, $\psi_{0}$ depends on the ionic strength of the subphase and the net charge density of the peptide monolayer, which is determined by the peptide’s sequence and the protonation state of its ionizable residues [41,42].

The surface potential ($\psi_{0}$) was calculated using the Gouy–Chapman–Graham equation for a planar interface:

(6) $$\sigma =\sqrt{8\varepsilon_{0}\varepsilon_{r}RTC_{b}}\sinh\left(\frac{F\psi_{0}}{2RT}\right)$$

where σ is the surface charge density, $\varepsilon_{0}$ is the vacuum permittivity, $\varepsilon_{r}$ is the relative permittivity of water, and $C_{b}$ is the bulk ionic concentration. This equation was solved numerically for $\psi_{0}$ using the estimated values of surface charge and subphase ionic strength for each condition. The resulting $\psi_{0}$ was then used to estimate the local interfacial pH according to Equation (5).

According to this model, assuming a monolayer charge density of +1.79${\mathrm{e/A}}_{p}$, the local pH may increase up to approximately 11. Although this is an approximate calculation, it highlights that in pure water, the interfacial pH can be substantially higher than the bulk pH (∼5.5), and consequently, the net monolayer charge is not maintained at +1.79${\mathrm{e/A}}_{p}$; instead, it approaches neutrality, such that the interface no longer attracts or repels ions. This promotes stronger intermolecular attractions and results in greater monolayer packing density, as evidenced by the lower $A_{p}$ (270 ± $5\;{Å}^{2}$) observed under these conditions. In the absence of the screening effect provided by salt, the balance of intramolecular electrostatic forces, including both repulsive and attractive interactions between oppositely charged residues present near the peptide’s isoelectric point (pH 6.67), would likely favor a more compact peptide conformation. It should be noted that, although the Gouy–Chapman model assumes a uniformly charged planar interface and thus does not fully capture the heterogeneity introduced by peptide adsorption onto a lipid monolayer, it provides a useful first-order approximation to assess qualitative trends in interfacial pH under different ionic strength conditions.

The presence of 150 mM NaCl expands significantly $A_{p}$ at all pH values tested compared with pure water. This substantial increase in $A_{p}$ suggests that the high ionic strength influences the intramolecular electrostatic interactions within the peptide. The calculations also revealed that, under physiological ionic strength, deviations between bulk and interfacial pH are markedly reduced, with the surface pH remaining close to 6.7 across all conditions (e.g., $pH_{s}\approx 6.7$ at $pH_{sb}$ = 5.5 and 7.5, and 6.9 at $pH_{sb}$ = 6.5).

The presence of NaCl can screen both repulsive interactions between like charges and attractive interactions (e.g., salt bridges) between oppositely charged residues. This screening effect can reduce the overall intramolecular electrostatic forces, allowing the peptide to adopt a more extended conformation at the interface, thereby occupying a larger molecular area.

Furthermore, the maximum $A_{p}$ appears to depend on the peptide’s net charge (q). The mean molecular area reaches its peak at pH 6.5 (370 ± $9\;{Å}^{2}$), precisely where the peptide’s net charge (q) is closest to neutrality (+0.47). In contrast, at pH 5.5, q is +1.79 and $A_{p}$ is $351\;{Å}^{2}$; similarly, at pH 7.5, q is -0.97 and $A_{p}$ is $351\;{Å}^{2}$. This pattern indicates that peptides, being polyelectrolytes, exhibit conformations highly sensitive to their charge state. When the peptide carries a significant net charge (either positive at pH 5.5 or negative at pH 7.5), intramolecular electrostatic forces induce a more compact monolayer, minimizing these repulsions, resulting in a smaller $A_{p}$. At pH 6.5, with a nearly neutral charge, the minimization of intramolecular electrostatic interactions allows the peptide to adopt a more extended conformation at the air-buffer interface, maximizing its molecular area. This finding provides direct evidence for pH-induced conformational changes in HMP1 at the interface. This has implications for understanding how the charge state dictates the effective “size” of a peptide at an interface.

An intriguing observation is the slight increase in $\pi_{c}$ from pH 6.5 to 7.5, despite a simultaneous decrease in $A_{p}$. Specifically, $A_{p}$ decreases from $370\;{Å}^{2}$ at pH 6.5 to $351\;{Å}^{2}$ at pH 7.5, yet $\pi_{c}$ increases 20.8 mN/m to 21.7 mN/m. If $A_{p}$ were the sole determinant of $\pi_{c}$, a decrease in $A_{p}$ (implying tighter initial packing) might be expected to lead to a higher $\pi_{c}$. However, the observed increase in $\pi_{c}$ while $A_{p}$ decreases suggests that factors other than initial packing density become dominant in determining the interface stability promoted by the peptide.

To assess the interfacial elasticity of the peptide film, the compressibility modulus ($C_{s}^{-1}$) was calculated as a function of surface pressure (Figure 1B). In pure water, the monolayer displays a relatively high compressibility modulus (approximately 45 mN/m), reflecting a relatively stiff film. This is in agreement with the high film density, suggesting the presence of electrostatic attractions between anionic and cationic residues.

In contrast, in the presence of 150 mM NaCl, the peptide carries a less negative or even positive charge at lower pH, leading to molecular packing and a reduced compressibility modulus. The modulus is lowest at pH 5.5, reflecting a more compressible monolayer. This may result from the peptide’s positive charge state and efficient screening of electrostatic interactions by salt, leading to increased flexibility. At pH 6.5 and 7.5, regardless of ionic strength, the monolayer becomes notably less compressible, as the peptide’s net charge approaches neutrality or becomes slightly negative. This minimizes both attractive and repulsive electrostatic forces, allowing for greater molecular flexibility at the interface.

Overall, these results demonstrate that the monolayer elasticity and mechanical properties depend not only on the net charge of the peptide but also on the local electrostatic environment, spatial arrangement of charged residues, and ionic strength. This highlights how subtle changes in environmental conditions can impact peptide organization and monolayer mechanics.

#### 3.1.2. Adsorption Characteristics of HMP1 at the Air-Water Interface

The surface activity of HMP1 was further investigated by monitoring its spontaneous adsorption onto a clean air-liquid interface, resulting in Gibbs adsorption isotherms. As shown in Figure 1C, the maximum change in surface pressure ($\Delta \pi_{max}$) is plotted as a function of peptide concentration added to subphase. The results indicate that the interface becomes saturated at a 1.0 µm peptide concentration under all subphase conditions tested. These values are similar to the saturation conditions of MP1 [14].

The ability of HMP1 to adsorb to the air-liquid interface and form a stable film is also influenced by subphase pH, as reflected by the maximum change in $\Delta \pi_{max}$ and the estimated mean molecular area per adsorbed peptide ($A_{ads}$) (Table 1). The highest $\Delta \pi_{max}$ at pH 6.5 (23.5 mN/m) coincides with the peptide’s net charge (q) being closest to zero (+0.47). $\Delta \pi_{max}$ directly quantifies the peptide’s efficiency in adsorbing to the air-liquid interface and reducing surface tension, reflecting the stability and density of the adsorbed film. Peptides generally adsorb more efficiently when their net charge is minimized because a high net charge (either positive or negative) can lead to electrostatic repulsion from the charged interface or between adsorbing peptide molecules, thereby hindering efficient adsorption and spreading. At pH 6.5, with a near-neutral charge, HMP1 experiences minimal electrostatic repulsion, facilitating optimal adsorption and the formation of a robust, stable film, hence the highest $\Delta \pi_{max}$. This aligns with the observation that $A_{p}$ is also maximal at pH 6.5, in line with a more extended conformation, facilitating better adsorption. This demonstrates that the charge state of HMP1 is a critical determinant of its adsorption efficacy at the air-liquid interface, with near-neutrality being most favorable for forming a strong interfacial film. This has implications for its function in environments with varying pH, such as biological membranes or drug delivery systems.

A notable discrepancy exists between $A_{p}$ and $A_{ads}$ values, particularly at pH 6.5. At this pH, $A_{p}$ is maximal ($370\;{Å}^{2}$), indicating a very expanded conformation at collapse, yet $A_{ads}$ is minimal ($238\;{Å}^{2}$), suggesting tight packing in the spontaneously adsorbed film or that the Gibbs films are not monomolecular films. This inverse relationship is significant. $A_{p}$ represents the area per molecule at the point of monolayer collapse during compression in a Langmuir trough experiment, while $A_{ads}$ is the area per molecule in a spontaneously adsorbed film, derived from adsorption kinetics. The substantial difference between $A_{p}$ and $A_{ads}$ implies that the peptide undergoes significant conformational adjustments depending on the experimental conditions (compression versus spontaneous adsorption). While the peptide might adopt a highly extended conformation when spread and compressed (leading to a high $A_{p}$), it can pack much more compactly when forming a stable adsorbed film (resulting in a low $A_{ads}$). The fact that $A_{ads}$ is smallest at pH 6.5, where $\Delta \pi_{max}$ is highest, suggests that the near-neutral charge state not only facilitates optimal adsorption but also allows the peptide to adopt a conformation that leads to the most effective and compact packing in the adsorbed layer.

This could involve “folding” or re-arranging upon adsorption to maximize intermolecular contacts and minimize interfacial area. This highlights the dynamic and adaptive nature of HMP1 at the interface. Its ability to undergo significant conformational changes to optimize packing and stability upon adsorption, particularly at its isoelectric point (or near-neutral charge), is crucial for its function and provides insights into the molecular mechanisms of peptide-interface interactions.

The compression behavior of MP1 monolayers has been previously studied on both water and NaCl solutions at pH 7.4 [14]. Similar to HMP1, the collapse pressure for MP1 was higher in NaCl (20 mN/m) than in water (17 mN/m). However, the $A_{p}$ values for MP1 were much smaller than those observed for HMP1, $150\;{Å}^{2}$ on water and $220\;{Å}^{2}$ in NaCl at pH 7.4, despite both peptides sharing a similar sequence framework except for the substitution of lysine (Lys) by histidine (His).

This substantial difference in molecular area cannot be attributed solely to the greater bulkiness of the histidine side chain compared with lysine. Instead, it is likely that other structural factors, such as peptide orientation at the interface and secondary structure content, play significant roles. For instance, a more tilted orientation or a lower propensity to form compact α-helical structures could increase the projected area per molecule at the interface. Circular dichroism studies indicated that HMP1 exhibits reduced α-helical content in solution at pH 5.5 and 7.5 compared with MP1 [18], supporting the idea that secondary structure differences contribute to the higher $A_{p}$ values observed for HMP1.

Furthermore, the presence of histidine, which is more pH-sensitive and can undergo charge state changes near physiological pH, may introduce additional conformational flexibility or favor less ordered structures at the interface. These factors, combined with possible differences in peptide tilt or partial insertion, likely account for the pronounced disparity in molecular area between the two peptides.

### 3.2. HMP1 Induced New Mixed Phase Formation in DPPC/HMP1 Monolayers

To investigate the interactions between HMP1 and a model lipid membrane component, films composed of pure DPPC lipid and DPPC co-spread with the peptide were prepared by carefully spreading their solutions onto the air-liquid interface of a Langmuir trough. The compression isotherm of the pure lipid monolayer exhibited a lift-off at approximately $100\;{Å}^{2}$/molecule, followed by a liquid-expanded (LE) to liquid-condensed (LC) phase transition plateau around 4 mN/m, extending from ∼80 to ∼$60\;{Å}^{2}$/molecule, and film collapse at 60 mN/m. These features are in excellent agreement with values previously reported in the literature [43].

**Figure 2:**
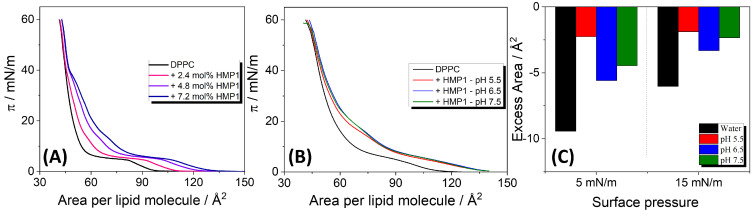
(**A**) π–*A* compression isotherms for pure DPPC and DPPC co-spread with different amounts of HMP1 onto water at 20 °C. The molecular area corresponds to the total monolayer area normalized by the number of lipids (not considering the peptide). (**B**) Compression isotherms for DPPC monolayers containing 7.2 mol% in saline solutions (NaCl 150 mM) at different pH and at 20 °C. (**C**) Excess area calculated from the mixed isotherms of DPPC + 7.2 mol% HMP1 shown in (**A**,**B**).

The compression isotherms (π-A) of DPPC co-spread with increasing molar fractions of HMP1 peptide (2.4, 4.8, and 7.2 mol%) on pure water (Figure 2A) revealed notable changes compared with the pure DPPC film. Specifically, a plateau appeared at surface pressures near the collapse pressure of the pure peptide film, as also evidenced at $C_{s}$^−1^ in Figure S1A. This strongly implies that at these higher pressures, peptide molecules are being squeezed out from the mixed monolayer into the subphase.

From the compression isotherms of the mixed films, the excess molecular area ($A_{ex}$) at fixed surface pressures was calculated, and the values are presented in Figure 2C. On pure water, consistently negative excess areas were observed across the tested peptide concentrations. A negative excess area indicates non-ideal mixing with peptide-lipid interactions more attractive than the interaction between the pure components, leading to a more compact mixed film than would be expected for an ideal mixture. These attractive interactions contribute to the stabilization of the mixed monolayer. In contrast, for MP1 on pure water, positive deviations from ideality were observed at all analyzed surface pressures, suggesting that peptide–lipid interactions are less attractive than peptide–peptide and lipid–lipid interactions [14].

In the presence of a saline solution, a negative $A_{ex}$ was also observed. In particular, although $A_{ex}$ was more negative at pH 6.5, indicating more favorable peptide-lipid mixing, the absolute molecular area of the mixed monolayer was larger than at pH 5.5 and 7.5. This suggests that while peptide–peptide interactions are less favorable at pH 6.5, resulting in less densely packed peptide monolayers, peptide–lipid interactions reach a maximum, thus inducing the most negative excess area under this condition.

Additionally, the isotherms overlap at 40 mN/m, indicating that HMP1 is squeezed out of the interface at this surface pressure, while at lower pressures, a fraction of peptide molecules remains within the monolayer. Previously, we demonstrated that the MP1 peptide is retained at the interface only up to surface pressures below 30 mN/m, both in pure water and under saline conditions [14]. Moreover, under the same saline conditions (MP1 in 150 mM NaCl, pH 7.4), no deviation from the additivity rule was observed, suggesting complete immiscibility. These results suggest that the histidine substitution enhances peptide–lipid interactions, potentially modulating membrane organization in a manner not observed for the native MP1 peptide.

**Figure 3:**
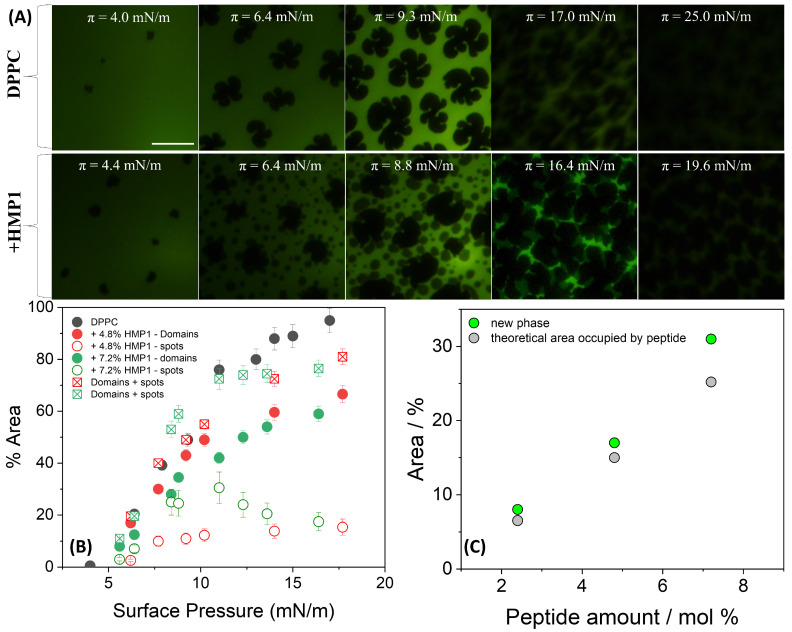
(**A**) Representative FM images for monolayers composed of DPPC or DPPC + 7.2 mol% peptide spread on pure water at indicated surface pressure and registered during compression at 20 °C. For the images obtained using FM, the monolayer contained 0.5 mole% of NBD-PC fluorescent dye. The scale bar represents 50 µm. (**B**) Area occupied by lipid domains (closed symbols) and by the new phase (’spots’, open symbols) at the indicated peptide concentration calculated using FIJI/ImageJ software (National Institutes of Health, Bethesda, MD, USA). The sum of the areas occupied by spots and LC domains is represented by square symbols. (**C**) Percentage of the area occupied by the new phase (green circles) and the theoretical area occupied by the peptide molecules (gray circles) calculated at 10–11 mN/m.

Fluorescence microscopy of DPPC films on pure water revealed the initial formation of small solid domains at low surface pressure (4 mN/m) (Figure 3A). As the compression progressed, these domains expanded into characteristic triskelion shapes, consistent with the coexistence plateau region. These morphological features are in good agreement with previously reported observations [14,44,45,46].

In the presence of peptide, the overall morphology of the LC domains remained largely unchanged; however, new peptide-induced microstructures, here referred to as ’spots’, appeared. A distinctive feature observed in the isotherms of the pure peptide, compared with those previously reported for MP1 [14], was the appearance of an inflection point in the 5–6 mN/m range (Figure 1A (inset)). At this surface pressure, small spots began to nucleate and coalesce, remaining closely associated with the edges of the LC domains. These spots likely could be a result of local phase separation or aggregation upon compression, reflecting partial miscibility and heterogeneous organization at the interface. As shown in Figure 3B, the total area occupied by the spots depends on peptide concentration primarily in the 7–12 mN/m pressure range. When the areas of the spots and LC domains are summed for both peptide concentrations, the total area closely matches the area of the LC domains in the absence of peptide, suggesting a conserved fraction of the interface covered by condensed structures.

This interpretation is supported by the fluorescence images (Figure 3A), which show that at similar surface pressures, LC domains remain present in both conditions; however, in the presence of peptide, their morphology appears more irregular, and numerous small dark spots are observed within the LE regions. These microstructures, absent in the peptide-free monolayer, likely reflect lateral heterogeneity and local restructuring induced by the peptide.

The total area occupied by these spots was slightly larger than the theoretical area [14] expected if they contained only the peptide molecules present at the interface (Figure 3C). This suggests that these microdomains are not exclusively peptide-rich. Given that the peptide was initially at the interface but tends to be excluded under high lateral pressure, it is plausible that this exclusion process perturbs the local lipid matrix. Such perturbation may promote the nucleation or stabilization of a new condensed lipid phase, potentially distinct from the classical LC domains, or induce local lipid reorientation. Thus, the observed spots likely represent reorganized, lipid-rich regions whose formation was triggered by peptide incorporation and subsequent exclusion during compression. This mechanism highlights the complex interplay between peptide presence, compression-induced molecular rearrangements, and lateral heterogeneity within the mixed monolayer, even in the absence of salt and electrostatic screening. In contrast, MP1 induced markedly different domain morphologies, characterized by more branched structures with longer and more curved arms compared with those formed in the absence of the peptide [14].

In saline solution (150 mM NaCl) at pHs 5.5, 6.5, and 7.5, the compression isotherms of pure DPPC were found to be similar to those obtained in pure water. This observation indicates that the subphase pH, within this physiological range and in the presence of a relevant salt concentration, exerts no significant effect on the intrinsic monolayer behavior of pure DPPC. Further confirmation of this pH-independence was provided by fluorescence microscopy, which showed that the subphase pH (5.5, 6.5, or 7.5) did not alter the characteristic triskelion-shaped solid domains (Figure S2). This stability is due to the zwitterionic lipids like DPPC, whose headgroup charge is largely pH-independent in this range. The consistency of DPPC behavior serves as a robust control, allowing for a clear attribution of observed pH-dependent effects in mixed films directly to the peptide.

**Figure 4:**
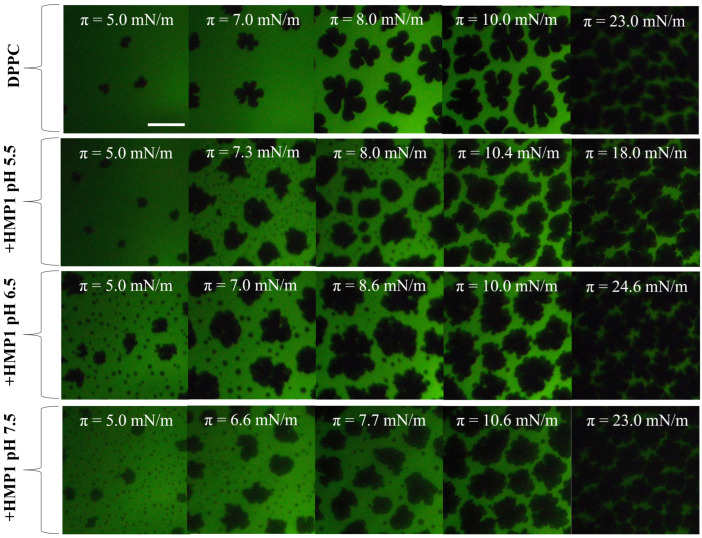
Representative FM images for monolayers composed of DPPC or DPPC + 7.2 mol% peptide spread on 150 mM NaCl at indicated surface pressure and subphase pH. The compression was performed at 20 °C. For the images obtained using FM, the monolayer contained 0.5 mole% of NBD-PC fluorescent dye. The scale bar represents 50 µm.

The introduction of HMP1 into the system led to a clear modification in the domain morphology of DPPC films, indicating direct interactions between the peptide and the lipid monolayer that alter the intrinsic self-assembly behavior of DPPC. In addition to the nucleation of typical liquid-condensed (LC) domains enriched in DPPC, a new phase appeared at approximately 5 mN/m, consistent with the formation of peptide-induced ’spots’. These microstructures were prominent at lower surface pressures, preferentially localized at the edges of the LC domains, even at low pressures (see Figure 4). This redistribution suggests that under saline conditions, the peptide actively modulates interfacial properties, promoting integration into existing lipid domains rather than forming isolated structures.

Domain boundaries represent regions of elevated interfacial energy due to line tension, and amphipathic peptides are known to preferentially associate with these regions, minimizing interfacial energy or exploiting packing defects. An increase in line tension typically drives domains to coalesce, reducing total boundary length and minimizing system energy. The observed incorporation of ’spots’ into the periphery of LC domains suggests that HMP1 modulates line tension or interfacial energy in such a way that favors the merging of these peptide-induced structures with pre-existing condensed lipid domains. This mechanism implies that, in the presence of NaCl, peptide localization at domain boundaries facilitates the integration of microdomains into larger LC domains, thereby stabilizing the monolayer structure and reducing the overall energetic cost associated with domain edges.

We hypothesized that the attractive interactions observed between peptide molecules on pure water are primarily mediated by the formation of salt bridges between aspartic acid and histidine residues. If all peptides are aligned in the same orientation at the interface, salt bridges may form between D2 and H4/H5, as well as between D8 and H11. Alternatively, if peptides adopt an antiparallel arrangement, salt bridges could form between D2 and H11 and between D8 and H4/H5. The establishment of these intermolecular interactions likely requires a specific peptide–peptide organization, which in turn facilitates favorable peptide–lipid interactions in mixed DPPC/peptide films.

Under conditions of high ionic strength (150 mM NaCl), charged residues on the peptide tend to bind counter-ions from the subphase, especially when the monolayer is in a more expanded state. As the film becomes more condensed, these counter-ions remain associated with the peptide, thereby weakening the direct interaction between aspartic acids and histidine residues. This reduction in peptide–peptide association leads to a decrease in the overall free energy of attractive interactions within the film. A similar phenomenon has been reported for the MP1 peptide; however, in the presence of 150 mM NaCl, no significant changes in domain morphology were detected, highlighting the unique role of histidine residues in HMP1 under these conditions.

Histidine residues, owing to their ${\mathrm{pK}}_{a}$ close to physiological pH, can undergo protonation–deprotonation transitions in response to subtle variations in a relevant pH-range, potentially inducing structural rearrangements within the amphipathic α-helix and altering peptide orientation at the interface. Such pH-driven conformational plasticity has been reported to regulate membrane binding affinity, insertion depth, and perturbation capacity of cationic antimicrobial peptides [14,18,19]. In our system, the lack of significant changes in the overall area fraction of condensed lipid phases suggests that HMP1 primarily affects local domain organization rather than inducing large-scale membrane condensation, an effect likely mediated by this pH-sensitive structural flexibility. These findings show that the histidine substitution enhances the strength and pH-responsiveness of peptide–lipid interactions, enabling HMP1 to remodel DPPC packing across pH 5.5–7.4 more effectively than MP1, with implications for membranes in mildly acidic or neutral environments.

The surface pressure associated with the structuring process is higher at pH 5.5 than at the other pH values (Figure 1A), and, as shown in Figure 4, the emergence of the new phase also occurs at higher surface pressure under these acidic conditions. This coincidence suggests a potential relationship between the onset of peptide conformational rearrangement and the nucleation of the new phase. Whether these spots are formed by reoriented peptide, reorganized lipid, or a combination of both remains an open question.

**Figure 5:**
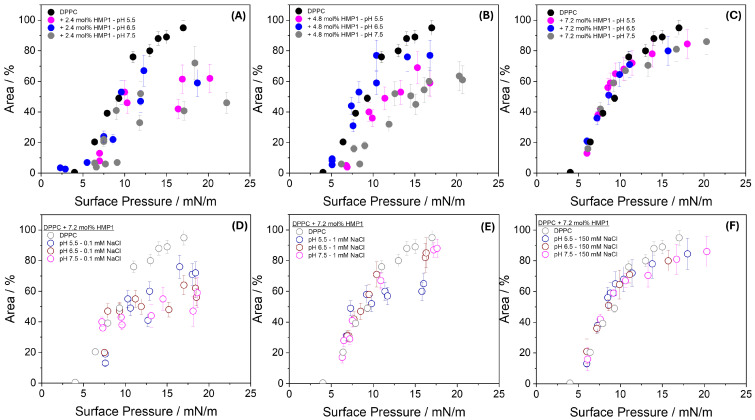
Area occupied by lipid domains plus the new phase (’spots’) at indicated pH in the absence and the presence of 2.4 mol% (**A**), 4.8 mol% (**B**), and 7.2 mol% (**C**) HMP1 as a function of surface pressure. Panel (**D**–**F**) shows the area occupied at 0.1 mM NaCl (**D**), 1 mM NaCl (**E**), and 150 mM NaCl (**F**), all at 7.2 mol% peptide and at the indicated pH.

To further quantify this effect, the combined area of the newly formed phase and the LC domains was calculated and compared with the LC phase area in pure DPPC monolayers, as shown in Figure 5A–C. The results showed that at lower peptide concentrations, the total area of the domains was smaller than that observed for pure DPPC across all pH conditions analyzed (Figure 5A,B). In contrast, at higher peptide concentration, the domain area became comparable to that of the pure lipid monolayer (Figure 5C). These findings indicate that the peptide–lipid interaction is both concentration- and pH-dependent, modulating the organization and stability of the LC domains. The higher surface pressure required for new phase formation at pH 5.5 may reflect the greater energetic barrier for peptide reorientation or exclusion when the peptide carries a higher net positive charge, thereby influencing the dynamics and extent of domain formation. Further studies, such as spectroscopic or molecular labeling techniques, would be needed to elucidate the precise composition and organization of these microdomains.

This behavior indicates that, under these conditions, peptide–lipid interactions reorganize the lateral structure of the monolayer, affecting the shape and organization of condensed domains without substantially changing the total area engaged in condensed structures. In other words, the presence of the peptide redistributes material at the interface, modulating domain morphology, but the overall amount of material participating in condensed phases remains comparable to that of the pure lipid monolayer.

To investigate the effect of ionic strength, experiments were conducted using subphases containing 0.1 mM and 1.0 mM NaCl at three different pH values Figure 5D–F, in addition to the conditions of pure water and 150 mM NaCl. At 0.1 mM NaCl (Figure 5D), the area occupied by condensed domains in the presence of peptide was consistently smaller than that of pure DPPC monolayers across all pH conditions, suggesting enhanced compaction or reorganization of the monolayer when electrostatic interactions remain largely unscreened. At 1.0 mM NaCl (Figure 5E), the domain area became comparable to that of pure DPPC at all pH values, except at pH 5.5, where it remained slightly smaller, possibly reflecting incomplete screening of the peptide’s net positive charge at this acidic pH. At 150 mM NaCl (Figure 5F), the domain areas closely matched those observed for pure DPPC and showed no significant pH dependence, indicating that strong ionic screening effectively suppresses differences arising from variations in peptide charge state.

These results demonstrate that ionic strength critically modulates peptide–lipid interactions at the interface. At very low ionic strength (0–0.1 mM), strong local electrostatic interactions dominate, leading to enhanced monolayer compaction. As ionic strength increases (1.0 mM), partial screening begins to mitigate these effects, and at physiological ionic strength (150 mM), the organization of the monolayer becomes largely insensitive to both pH and peptide charge state. This highlights the essential role of ionic environments in regulating peptide–membrane interactions and suggests that biological membranes may rely on ionic conditions to buffer the structural effects of charged peptides. The peptide-induced domains observed here reflect lateral heterogeneity arising from specific molecular interactions at the membrane interface.

Additionally, these morphological changes differ markedly from those previously reported for the MP1 peptide, which contains lysine instead of histidine. In pure water, MP1 co-crystallizes with DPPC, promoting the formation of elongated domains, while under saline conditions (150 mM NaCl), MP1 remains within the liquid-expanded (LE) phase and does not perturb LC domains, being excluded into the subphase upon reaching its collapse pressure. In contrast, HMP1 continues to associate with the monolayer and promotes the formation of small condensed domains even in high-salt conditions [14].

This suggests that the lysine-to-histidine substitution profoundly affects the peptide’s interfacial behavior and its ability to modulate lipid organization. Given that histidine has a lower pKa and is more sensitive to local pH and environment than lysine, this substitution likely alters the peptide’s charge distribution and capacity for specific interactions with the lipid matrix. Thus, this behavior emphasizes how subtle changes in peptide sequence and environmental conditions can modulate peptide–membrane interactions, phase behavior, and lateral organization, insights that are directly relevant for understanding peptide function in complex biological membranes.

### 3.3. Peptide Insertion at Constant Area: Sensitivity to Membrane Composition and Order

To further explore the selectivity of HMP1 interactions with lipid interfaces under conditions that mimic physiological lateral pressures, we conducted constant-area insertion experiments using different lipid monolayers and compositions.

Insertion experiments at constant area were conducted at a lateral pressure of 30 mN/m, a condition that approximates the lipid density in biological membranes. As shown in Table 3, HMP1 did not induce any significant surface pressure change in pure POPC monolayers at any pH tested, confirming its low affinity for fluid, disordered membranes. In contrast, substantial surface pressure increases were detected for monolayers containing cholesterol or saturated lipids. Notably, the lysine-containing peptide MP1 showed no detectable insertion under identical conditions for POPC, DPPC, or PC/Chol monolayers, underscoring the functional consequences of the lysine-to-histidine substitution [16].

These findings reinforce the biological relevance of HMP1’s selective interaction profile. The ability of HMP1 to interact more strongly with cholesterol-containing and ordered lipid domains suggests a potential preference for raft-like microdomains in biological membranes. The selective affinity of HMP1 for these domains may reflect a mechanism for targeting specific membrane environments. Moreover, the absence of interaction with fluid POPC-rich regions and the lack of insertion by MP1 under identical conditions emphasize how subtle sequence modifications modulate membrane selectivity, lateral organization, and potentially biological function.

Regarding the effect of pH, HMP1 causes higher perturbations to 3POPC/1Chol monolayers at pH 6.5 compared with 7.5. From a biological perspective, this ability to modulate membrane lateral organization in a pH- and salt-dependent manner may be particularly relevant in vivo, where variations in ionic strength and pH occur in specific microenvironments such as inflamed tissues, endosomal compartments, or tumor regions [23].

This observation points to the potential pH-selective behavior of HMP1 and underscores its relevance for targeting tumor tissues. However, to fully explore the selectivity and therapeutic potential of HMP1, additional studies using lipid compositions that more closely mimic the membranes of normal and cancer cells are needed.

## 4. Conclusions

This study demonstrates that the adsorption and packing behavior of HMP1 at the air–liquid interface is strongly influenced by pH and ionic strength, reflecting the sensitivity of its conformation and interfacial organization to charge state and environmental conditions. When mixed with DPPC, HMP1 induces significant morphological changes, including the formation of peptide-associated microdomains, even though the overall area occupied by condensed phases remains comparable to that of pure DPPC monolayers. This indicates that HMP1 reorganizes lateral structure without substantially altering the fraction of the interface engaged in condensed domains.

Constant-area insertion experiments further revealed that HMP1 preferentially interacts with ordered or cholesterol-rich membranes under physiologically relevant lateral pressures, while showing negligible interaction with fluid POPC membranes. The contrasting behavior of the lysine-containing peptide MP1, which did not induce similar morphological changes or membrane insertion under identical conditions, underscores the critical impact of the lysine-to-histidine substitution on interfacial behavior and lipid organization. Furthermore, HMP1 exhibited pH-dependent interactions with cholesterol-containing monolayers, a property that deserves further investigation given its potential selectivity toward cancer cells.

Together, these results highlight the ability of HMP1 to modulate membrane structure in a manner dependent on lipid composition, membrane order, pH, and ionic strength, suggesting that even subtle changes in peptide sequence can confer selective targeting of specific membrane environments. These findings provide valuable insights for understanding peptide–membrane interactions and may inform the design of peptide-based therapeutics targeting distinct membrane microdomains.

## Figures and Tables

**Table 1:** Physico-chemical features of HMP1 dependent on pH: mean molecular area at higher lateral packing ($A_{p}$), pressure at peptide’s film highest compressibility modulus ($\pi_{c}$), maximum change in surface pressure due to peptide adsorption ($\Delta \pi_{max}$), and the area per peptide molecule after adsorption onto a clean interface ($A_{ads}$).

Subphase Condition	$A_{p}^{\;a}$ (${Å}^{2}$)	$\pi_{c}^{\;a}$ (mN/m)	$\Delta \pi_{\max}^{\;b}$ (mN/m)	$A_{\mathrm{ads}}^{\;c}$ (${Å}^{2}$)
Water, pH ∼5.5	270 ± 5	17.2 ± 0.2	22.3 ± 0.9	248 ± 19
150 mM NaCl, pH 5.5	351 ± 8	18.3 ± 0.9	20.4 ± 1.0	274 ± 15
150 mM NaCl, pH 6.5	370 ± 9	20.8 ± 0.6	23.5 ± 1.2	238 ± 13
150 mM NaCl, pH 7.5	351 ± 1	21.7 ± 0.2	22.0 ± 1.0	248 ± 4

^*a*^ Values obtained from Figure 1A. ^*b*^ Values obtained from Figure 1C. ^*c*^ Values obtained from adsorption experiments using Equation (2).

**Table 2:** Net charge (q) of the peptide HMP1. The contribution of each residue, histidine (His), aspartic acid (Asp), and N-terminal amine (${\mathrm{NH}}_{2}$), was calculated according to the Henderson–Hasselbalch equation under the indicated pH conditions.

pH	q	${\mathrm{NH}}_{2}$	His	Asp
pH 5.5	+1.79	+1.00	+2.73	−1.94
pH 6.5	+0.48	+0.97	+1.50	−1.99
pH 7.5	−0.97	+0.76	+0.27	−2.00

**Table 3:** Change in surface pressure due to peptide insertion into lipid monolayer at 30 mN/m.

Lipid Composition	pH 5.5 (mN/m)	pH 6.5 (mN/m)	pH 7.5 (mN/m)
POPC	negligible	negligible	negligible
3POPC/1Chol	6.0 ± 0.8	7.5 ± 1.0	6.7± 1.0
DPPC	4.1 ± 1.0	3.3 ± 0.8	4.9 ± 0.7

## Data Availability

The data presented in this study are available in this article and Supplementary Materials.

## Supplementary Materials

The following supporting information can be downloaded at: https://www.mdpi.com/article/10.3390/pharmaceutics17101287/s1, Figure S1: Compressibility modulus of pure DPPC and DPPC/peptide monolayers, highlighting the surface pressure at which peptide exclusion from the interface occurs; Figure S2: FM images show no change in the phase morphology of pure DPPC monolayers under varying subphase pH and ionic strength.
