# Supplementary material for: Tweaking Polybia-MP1: How a Lysine-Histidine Swap Redefines Its Surface Properties
Source: Pharmaceutics. 2025 Oct 2;17(10):1287. doi: 10.3390/pharmaceutics17101287 (PMC12567272; doi:10.3390/pharmaceutics17101287)
Supplement: Supplementary file 1 [file pharmaceutics-17-01287-s001.zip › pharmaceutics-3798218-supplementary.pdf]

Tweaking Polybia-MP1: How a Lysine-Histidine Swap Redefines Its Surface Properties

Kenneth M. F. Miasaki[1], Bibiana M. Souza[2], Mario S. Palma[2], Natalia Wilke[3,4], João Ruggiero Neto[1]* and Dayane S. Alvares[1]*.

[1] Department of Physics, IBILCE, UNESP - São Paulo State University, São José do Rio Preto, 15054-000, SP, Brazil.

[2] Department of Basic and Applied Biology, Institute of Biosciences, UNESP - São Paulo State University, Rio Claro, 13506-900, SP, Brazil.

[3] Centro de Investigaciones en Química Biológica de Córdoba (CIQUIBIC), CONICET, Haya de la Torre y Medina Allende, Ciudad Universitaria, Córdoba, X5000HUA, Argentina.

[4] Departamento de Química Biológica Ranwel Caputto, Facultad de Ciencias Químicas, Universidad Nacional de Córdoba, Córdoba, X5000HUA, Argentina.

Corresponding authors*: dayane.alvares@unesp.br, joao.ruggiero@unesp.br.

Orcid: KMFM: 0000-0001-7190-9148, DSA: 0000-0002-6521-9148, BMS: 0000-0002-4355-2361, MSP: 0000-0002-7363-8211, JRN: 0000-0002-2283-3316

Supplementary Material

Figure S1 - Compressibility modulus of pure DPPC and DPPC/peptide monolayers, highlighting the surface pressure at which peptide exclusion from the interface occurs.

Figure S2 - FM images show no change in the phase morphology of pure DPPC monolayers under varying subphase pH and ionic strength.

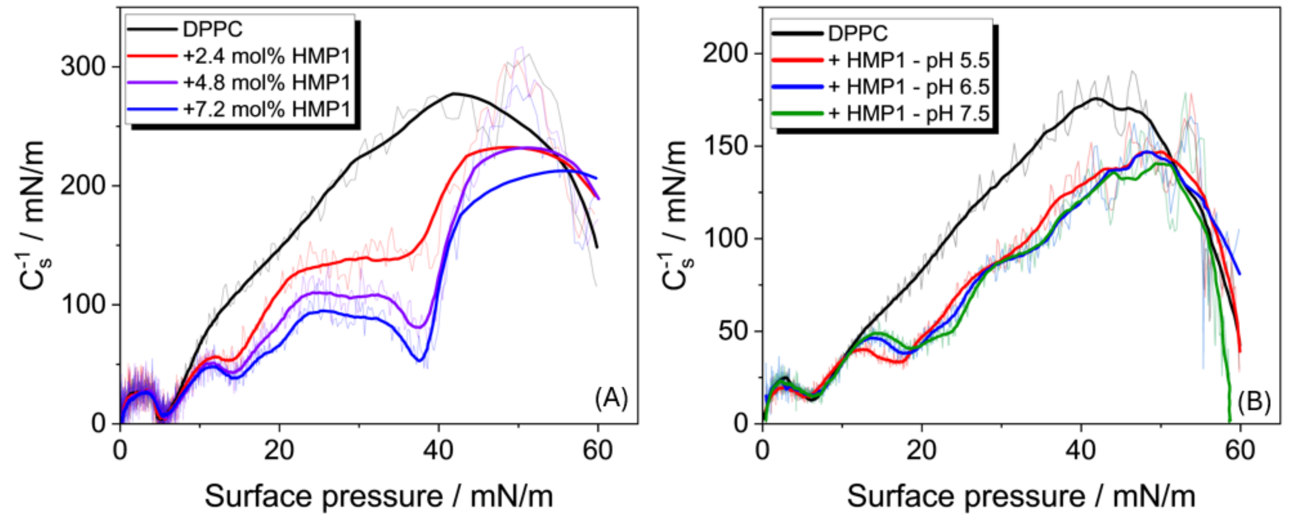

Figure S1: Compressibility modulus for pure DPPC and (A) DPPC co-spread with increasing amounts of HMP1 on pure water, calculated from the isotherms shown in Fig. 2A; and (B) DPPC co-spread with 7.2 mol% HMP1 in 150 mM NaCl at different pH values calculated from Fig. 2B.

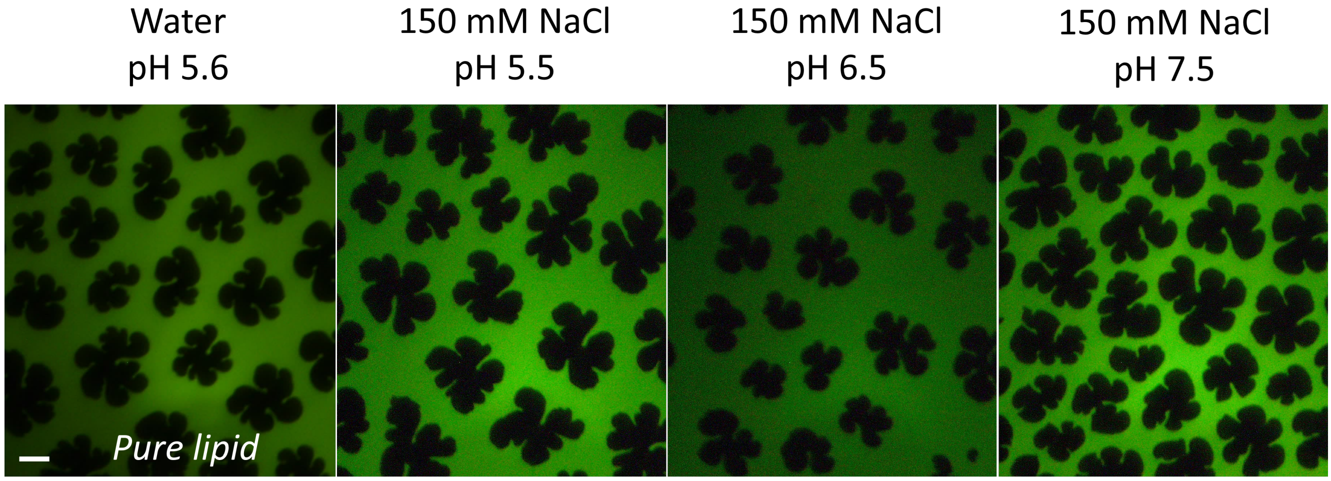

Figure S2: Representative FM images of monolayers composed of pure DPPC at ∼8 mN/m, acquired at the indicated subphase pH and ionic strength conditions. Compression experiments were performed at 20 °C. For FM imaging, the monolayers contained 0.5 mol% NBD-PC fluorescent dye. Scale bar: 20 μm.
